# Effectiveness of early treatment with plasma exchange in patients with Stevens–Johnson syndrome and toxic epidermal necrolysis

**DOI:** 10.1038/s41598-024-53653-5

**Published:** 2024-02-05

**Authors:** Atsushi Senda, Kiyohide Fushimi

**Affiliations:** 1 Department of Acute Critical Care and Disaster Medicine, Graduate School of Medical and Dental Sciences, Tokyo Medical and Dental University, 1-5-45 Yushima, Bunkyo-Ku, Tokyo, 113-8510 Japan; 2 Department of Health Policy and Informatics, Graduate School of Medical and Dental Sciences, Tokyo Medical and Dental University, 1-5-45 Yushima, Bunkyo-Ku, Tokyo, 113-8510 Japan

**Keywords:** Skin diseases, Medical research

## Abstract

Stevens–Johnson syndrome and toxic epidermal necrolysis (SJS/TEN) are potentially fatal medical conditions that lack established treatment. Therapeutic plasma exchange (PE) is a potential treatment option; however, its effectiveness is unclear. We aimed to evaluate the effectiveness of PE in patients with SJS/TEN. A retrospective cohort study was conducted using data from the Japanese National Administrative Claims database from 2016 to 2021. The analysis included 256 patients diagnosed with SJS/TEN who were admitted to the intensive care unit, of whom 38 received PE and 218 did not. The outcomes of patients who did and did not receive PE within the first 24 h of admission were compared. The risk ratios and 95% confidence intervals of the PE group compared with those of the no-PE group were as follows: in-hospital mortality, 0.983 (0.870–1.155); 30-day mortality rate, 1.057 (0.954–1.217); 50-day mortality rate, 1.023 (0.916–1.186); and length of hospital stay, 1.163 (0.762–1.365). This study does not provide evidence of a benefit of PE in reducing mortality or length of hospital stay in patients with severe SJS/TEN.

## Introduction

Stevens–Johnson syndrome and toxic epidermal necrolysis (SJS/TEN) are considered part of a single spectrum of diseases. Although uncommon, this condition constitutes a dermatologic emergency owing to its severe manifestations and potentially fatal outcome^1,2^. The mainstay of management is supportive treatment, which includes discontinuation of causative agents, fluid replacement therapy, local treatment, nutritional support, and respiratory management^3^. Active anti-inflammatory therapy based on its pathophysiology is often administered simultaneously. Several pharmacologic treatment options have been proposed, including corticosteroids^4^, cyclosporine^5–7^, etanercept^8^, and intravenous immunoglobulin (IVIG)^6^. However, their effectiveness remains unclear owing to the rarity of the disease.

Therapeutic plasma exchange (PE) is another treatment option but evidence of its effectiveness is limited to case reports and small case series^9–11^. The rationale for PE in SJS/TEN is to filter causative drugs and their metabolites, and remove immunological factors involved in the disease. However, conflicting results have been reported^12^, and its effectiveness remains unknown. The objective of this study was to assess the effectiveness of PE in patients with SJS/TEN.

## Methods

### Study design, setting, and data sources

To assess the effectiveness of PE in patients with SJS/TEN, we conducted a retrospective cohort study using the Japanese Diagnosis Procedure Combination (DPC) database. This database is a comprehensive classification system designed to support a prospective payment model for acute-phase inpatient hospital care, which involved more than 1700 hospitals in 2020. This database contains hospital identification codes and demographic information, such as sex, age, weight, date of admission, scores reflecting activities of daily living, status on admission and discharge, and post-admission complications. Additionally, the database records all medications, procedures, and care provided during each patient’s hospital stay. Diagnoses were documented using appropriate codes from the International Classification of Diseases, 10th edition (ICD-10). Further details of this database have been reported previously^13,14^.

### Ethical approval and consent to participate

The study was approved by the Institutional Review Board of the Tokyo Medical and Dental University (#788) and the requirement for informed consent was waived owing to the retrospective nature of the study and the use of anonymised data. The study was conducted in accordance with the Declaration of Helsinki.

### Study population

The study included patients with SJS/TEN admitted to hospitals participating in the DPC between 1 April 2016 and 31 March 2021. The inclusion criteria were patients diagnosed with SJS, TEN, or SJS/TEN (ICD codes: L51.1, L51.2, and L51.3) admitted to an intensive care unit. This restriction was imposed to specifically assess the effectiveness of PE in the most severe cases that are most likely to derive benefit from this treatment. Patients with missing data on any of the analysed variables, aged younger than 16 years, pregnant women, and patients discharged within the first 2 days after admission were excluded. Additionally, patients who received etanercept or cyclosporine were excluded because of the small sample size, making covariate adjustment difficult.

### Definitions and outcomes

The PE therapy group comprised patients who received PE within the first 24 h after admission. The primary outcome was in-hospital mortality. Secondary outcomes were 30-day mortality, 50-day mortality, length of hospital stay, and onset of sepsis during the hospital stay.

### Statistical analysis

An in-hospital mortality risk adjustment model was developed using a random sample of 70% of the cohort. The covariables included age, sex, Charlson Comorbidity Index, diabetes, heart failure acute lung injury, level of consciousness (alert or not), gross wound-treated area, use of mechanical ventilation, renal replacement therapy, administration of noradrenaline, dobutamine, and blood cell transfusion, and type of hospital (academic or not), were chosen based on their clinical relevance^15^. Concurrent treatment with hydrocortisone and IVIG was also included as covariates. The remaining 30% of the cohort was used to validate the prognostic accuracy of the model using the area under the receiver operating characteristic curve (AUROC) and the Hosmer–Lemeshow goodness-of-fit test. The treatment effect was inferred using doubly robust estimation^16^ using logistic regression analysis with the same covariates as in the original analysis. Weight trimming of the propensity score was minimally applied (1%), according to methods described previously^17,18^. The confidence interval was estimated using bootstrapping with 3,000 replications. All statistical analyses were performed using Python (version 3.8.6).

## Results

**Figure 1:**
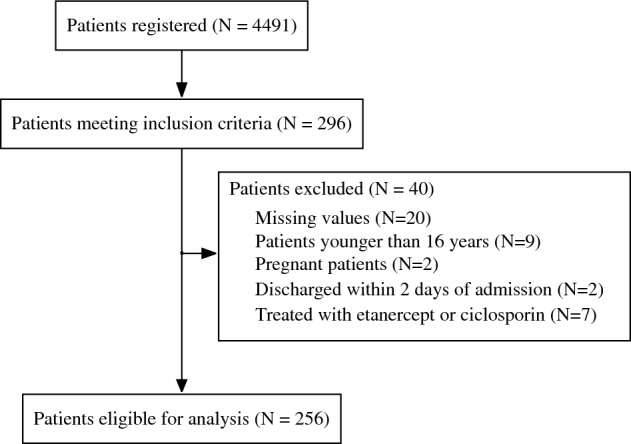
Flow diagram showing patient selection.

Figure 1 shows a flowchart of the patient selection process. During the study period, 4,491 patients were admitted to DPC-participating hospitals with SJS/TEN, of whom 296 were admitted to intensive care units and met the inclusion criteria. After excluding 40 patients with one or more exclusion criteria, 256 patients were included in the analysis. Among them, 38 underwent PE, of whom 34 patients were treated with PE only, and 4 patients received treatment with PE in combination with corticosteroids and/or IVIG within the first 2 days of admission. The median number of PE treatments were 3 (interquartile range 2–5). Table 1 compares the patient characteristics, and Table 2 compares the outcomes of the PE and no-PE groups. The unadjusted outcomes of the combination of PE, corticosteroids or IVIG are shown in the Supplementary Table S1. The overall in-hospital mortality rate was 15.6% (40/256), and the 30-day and 50-day mortality rates were 7.4% (19/256) and 11.3% (29/256), respectively. The median hospital stay was 31 days (interquartile range, 20–54 days). The established model demonstrated good calibration for the validation cohort, with an AUROC of 0.86 and a Hosmer–Lemeshow p = 0.91 (Supplementary Fig. S1).

**Table 1:** Patient characteristics according to whether they were treated with plasma exchange.

Patient characteristic	Received plasma exchange	Did not receive plasma exchange
Number of patients, n	38	218
Age (years), median [IQR]	61 [42–74]	65 [54–78]
Sex, female, n (%)	18 (47.39)	104 (47.71)
Charlson Comorbidity Index, median [IQR]	0 [0–2]	1 [0–2]
Consciousness, alert, n (%)	29 (76.32)	124 (56.89)
Mechanical ventilation use, n (%)	8 (21.05)	60 (27.52)
Renal replacement therapy, n (%)	10 (26.32)	24 (11.00)
Acute lung injury, n (%)	2 (5.26)	6 (2.75)
Diabetes, n (%)	6 (15.79)	36 (15.51)
Red blood cell transfusion, n (%)	16(42.11)	83 (38.1)
Heart failure, n (%)	2 (6.26)	15 (6.88)
Immunoglobulin administration, n (%)	6 (15.79)	103 (47.25)
Dobutamine administration, n (%)	3 (7.89)	18 (8.26)
Noradrenaline administration, n (%)	18 (47.37)	72 (33.03)
Hydrocortisone administration, n (%)	1 (2.63)	25 (11.47)
Gross wound-treated area/day, cm^2^, median [IQR]	5990 [3240–6590]	4825 [2225–5820]
Treated in academic hospital, n (%)	26 (68.42)	92 (42.20)

*IQR* interquartile range.

**Table 2:** Unadjusted outcomes in patients who did and did not receive plasma exchange.

Outcome	Received plasma exchange	Did not receive plasma exchange
In-hospital mortality (%)	18.42	11.30
30-day mortality (%)	13.16	6.50
50-day mortality (%)	15.79	10.57
Length of hospital stay (days) [IQR]	37 [21.0–59.5]	28 [17.5–52.5]

*IQR* interquartile range.

**Figure 2:**
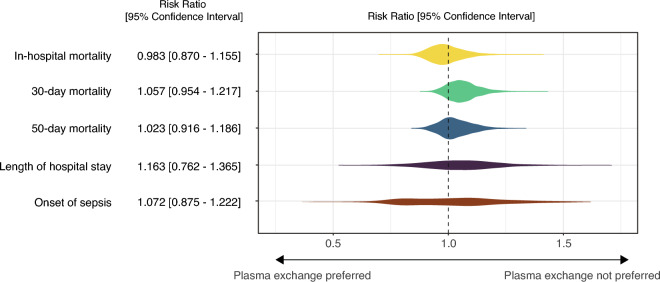
Summary of the outcomes comparing patients treated with and without early plasma exchange after adjustment for covariates. The risk ratios were adjusted for age, sex, Charlson Comorbidity Index, diabetes, heart failure, acute lung injury, level of consciousness (alert or not), gross wound-treated area, use of mechanical ventilation, renal replacement therapy, administration of noradrenaline, dobutamine, and blood cell transfusion, and type of hospital (academic or not).

After adjustment, the risk ratios for PE treatment compared with no PE were 0.983 (95% CI 0.870–1.155) for in-hospital mortality, 1.057 (95% CI 0.954–1.217) for 30-day mortality, 1.023 (95% CI 0.916–1.186) for 50-day mortality , 1.163 (95% CI 0.762–1.365) for the length of hospital stay, and 1.072 (95% CI 0.875–1.222) for the onset of sepsis during the hospital stay (Fig. 2).

In a subgroup analysis excluding patients who received hydrocortisone or IVIG, the risk ratio for in-hospital mortality in patients who received PE compared with those with no PE was 0.982 (95% CI 0.723–1.233) (Supplementary Fig. S2).

## Discussion

The effectiveness of initiating PE immediately on hospital admission in patients with severe SJS/TEN was evaluated using a nationwide database and adjusted using a doubly robust estimation method to minimise confounding. The results showed no benefit of PE for reducing in-hospital mortality or the length of hospital stay.

A recent study compared the effectiveness of PE with IVIG treatment and found no observable benefits of PE over IVIG in patients who did not respond effectively to systemic corticosteroid treatment^19^. However, the effectiveness of IVIG treatment also remains unclear, and so it is unclear whether PE and IVIG are equally effective or equally ineffective for treating SJS/TEN. Furthermore, as the effect of corticosteroids on patients with SJS/TEN has not been definitively established, it is unclear whether the study included patients with severe SJS/TEN resistant to corticosteroid treatment, or whether treatment with corticosteroids affected disease progression.

Several studies have shown effectiveness of PE in patients with SJS/TEN, but they were either small case series or individual case reports^9–12^. Consequently, various biases, including selection, survivorship, and publication biases, were present in these studies. The rationale for PE lies in its ability to remove toxins, including drugs, drug metabolites, and other cytotoxic mediators^11,20–23^. However, PE also has some potential disadvantages. PE may deplete immunoglobulin levels, potentially increasing the risk of sepsis^24^. Additionally, early initiation of PE may disrupt the optimal timing for other potentially effective treatments^19^. This study, with appropriate adjustments for concurrent treatment, provided no evidence that PE reduced mortality or shortened the length of hospital stay. Additionally, the study revealed that patients who received PE did not have an increased risk of sepsis.

This study has several limitations. First, as it was a retrospective study, the validation of the diagnostic data in the database may not be as exhaustive as that of a purpose-designed prospective study. However, available evidence suggests that the diagnostic specificity of the DPC database exceeds 96%^25^. Second, patients who were discharged within 2 days were excluded, creating the possibility of selection bias. Third, some patients were treated with a combination of PE and other treatments, such as corticosteroids and IVIG. We were unable to perform a subgroup analysis according to the types of co-treatment received because of the small number of patients, so were unable to assess whether effect modification was present. Fourth, the potential for residual confounding remains because of the retrospective study design and the lack of data on vital signs and laboratory test results. Consequently, SCORTEN—a widely used general severity score^26^ for SJS/TEN—was not available in this study. Nonetheless, our predictive model demonstrated comparable accuracy to that of the SCORTEN score. Fifth, because the number of days from the onset of illness to hospital admission could not be determined, the stage of the illness at which patients received PE remains unclear. Finally, the study was conducted based on the assumption that if PE is to benefit patients, the most severe cases of SJS/TEN are the ones that are most likely to benefit. The effectiveness of PE should be evaluated in patients with a range of disease severity so that the results can be generalised to all cases of SJS/TEN.

In contrast to previous studies^9–11^, this study did not find any evidence of a benefit of early PE in patients with severe SJS/TEN.

### Supplementary Information

Supplementary Figure S1.

Supplementary Figure S2.

Supplementary Table S1.

## Data Availability

The datasets generated during and/or analysed during the current study are available from the corresponding author on reasonable request.
