# Supplementary material for: Effectiveness of early treatment with plasma exchange in patients with Stevens–Johnson syndrome and toxic epidermal necrolysis
Source: Sci Rep. 2024 Feb 5;14:2893. doi: 10.1038/s41598-024-53653-5 (PMC10844598; doi:10.1038/s41598-024-53653-5)
Supplement: Supplementary file 1 — Supplementary Figure S1. [file 41598_2024_53653_MOESM1_ESM.pdf]

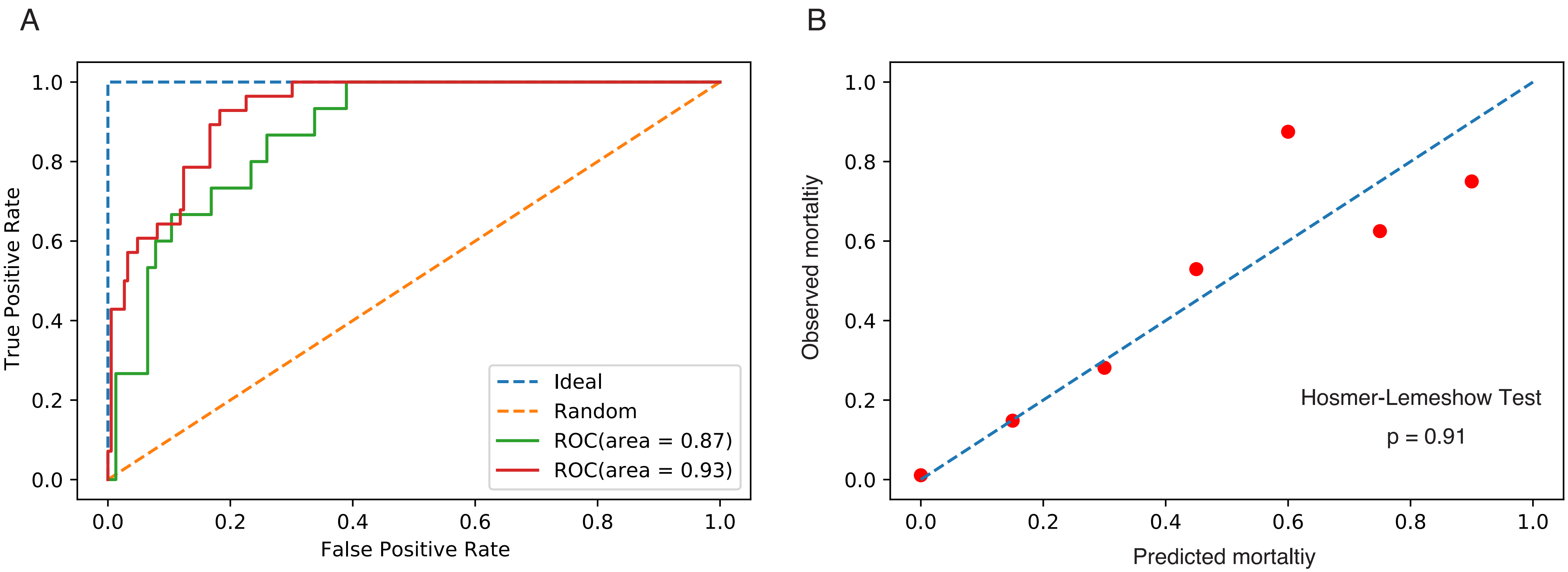

Supplementary Figure 1: Evaluation of the case-mix classification model's suitability.
(A) ROC curves in the validation cohort using the risk-adjustment model. (B) Hosmer-Lemeshow goodness-of-fit test. Abbreviations: ROC, receiver operating characteristic
