# Supplementary material for: Effectiveness of early treatment with plasma exchange in patients with Stevens–Johnson syndrome and toxic epidermal necrolysis
Source: Sci Rep. 2024 Feb 5;14:2893. doi: 10.1038/s41598-024-53653-5 (PMC10844598; doi:10.1038/s41598-024-53653-5)
Supplement: Supplementary file 2 — Supplementary Figure S2. [file 41598_2024_53653_MOESM2_ESM.pdf]

	Risk Ratio [95% Confidence Interval]	Risk Ratio [95% Confidence Interval]
In-hospital mortality	0.982 [0.723 - 1.233]	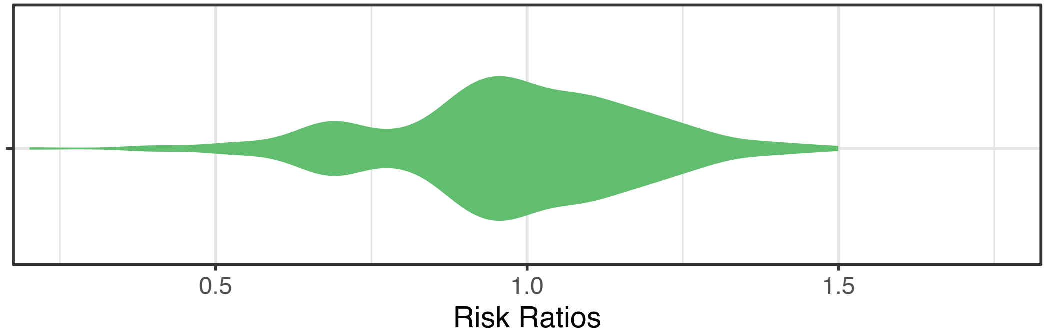

Plasma exchange preferred ← → Plasma exchange not preferred

Supplementary Figure 2: Subgroup analysis on the risk of in-hospital mortality comparing patients treated with and without early plasma exchange after adjustment for covariates excluding patients treated with hydrocortisone or IVIG. The risk ratios were adjusted for age, sex, Charlson Comorbidity Index, diabetes, heart failure, acute lung injury, level of consciousness (alert or not), gross wound-treated area, use of mechanical ventilation, renal replacement therapy,administration of noradrenaline, dobutamine, and blood cell transfusion, and type of hospital (academic or not).
