# Supplementary material for: Effectiveness of early treatment with plasma exchange in patients with Stevens–Johnson syndrome and toxic epidermal necrolysis
Source: Sci Rep. 2024 Feb 5;14:2893. doi: 10.1038/s41598-024-53653-5 (PMC10844598; doi:10.1038/s41598-024-53653-5)
Supplement: Supplementary file 3 — Supplementary Table S1. [file 41598_2024_53653_MOESM3_ESM.docx]

Supplementary Table S1. Unadjusted outcomes of patient subgroups who did and did not receive plasma exchange

| Patient subgroups | In hospital mortality (%) |
| --- | --- |
| **Received plasma exchange** |  |
| Received hydrocortisone | 50.00 |
| Did not receive hydrocortisone | 14.70 |
| Received immunoglobulin | 16.66 |
| Did not receive immunoglobulin | 21.43 |
| **Did not receive plasma exchange** |  |
| Received hydrocortisone | 30.43 |
| Did not receive hydrocortisone | 13.33 |
| Received immunoglobulin | 22.54 |
| Did not receive immunoglobulin | 8.62 |
